# Adult hippocampal neurogenesis inversely correlates with microglia in conditions of voluntary running and aging

**DOI:** 10.3389/fnins.2013.00145

**Published:** 2013-08-20

**Authors:** Elias Gebara, Sebastien Sultan, Jacqueline Kocher-Braissant, Nicolas Toni

**Affiliations:** Department of Fundamental Neurosciences, University of LausanneLausanne, Switzerland

**Keywords:** adult neurogenesis, dentate gyrus, microglia, running, aging

## Abstract

Adult hippocampal neurogenesis results in the formation of new neurons and is a process of brain plasticity involved in learning and memory. The proliferation of adult neural stem or progenitor cells is regulated by several extrinsic factors such as experience, disease or aging and intrinsic factors originating from the neurogenic niche. Microglia is very abundant in the dentate gyrus (DG) and increasing evidence indicates that these cells mediate the inflammation-induced reduction in neurogenesis. However, the role of microglia in neurogenesis in physiological conditions remains poorly understood. In this study, we monitored microglia and the proliferation of adult hippocampal stem/progenitor cells in physiological conditions known to increase or decrease adult neurogenesis, voluntary running and aging respectively. We found that the number of microglia in the DG was strongly inversely correlated with the number of stem/progenitor cells and cell proliferation in the granule cell layer. Accordingly, co-cultures of decreasing neural progenitor/glia ratio showed that microglia but not astroglia reduced the number of progenitor cells. Together, these results suggest that microglia inhibits the proliferation of neural stem/progenitor cells despite the absence of inflammatory stimulus.

## Introduction

In the mammalian brain, adult neural stem cells reside in the subventricular zone and subgranular zone (SGZ) of the hippocampus and continue to produce new neurons throughout life (Altman, 1969). Adult neurogenesis has been observed in all mammalian species including humans (Eriksson et al., 1998) and results in the formation of new neurons in the olfactory bulb and the dentate gyrus (DG) of the hippocampus. Radial glia-like (RGL) neural stem cells that reside in the SGZ of the DG, proliferate and give rise to transit-amplifying progenitors (TAP) expressing the T-box brain gene 2 (Tbr2) antigen which then give rise to doublecortin (DCX)-expressing immature neurons (Gage, 2000; Yao et al., 2012). After a phase of maturation, newly-formed neurons express the mature neuronal marker Neu N, functionally integrate into the hippocampal network (Toni and Sultan, 2011) and participate to mechanisms of learning and memory (Aimone et al., 2010). Each of these steps is highly regulated by signaling from the neurogenic niche, and an increasing number of pro-neurogenic factors are being discovered, with great potential interest for cell-replacement therapeutic approaches. The neurogenic niche is constituted by stem cells and their progenies, astrocytes, oligodendrocytes, endothelial cells, microglia, mature and immature neurons (Shihabuddin et al., 2000; Song et al., 2002; Zhao et al., 2008; Bonaguidi et al., 2011). These cells release a wide array of factors that control adult neurogenesis. However, the contribution of specific cell types on the processes of adult neurogenesis remains poorly determined.

Of particular interest, microglia are abundant and widely distributed throughout the adult brain. These cells are involved in the inflammatory reaction and act as the resident immune cells of the brain. Their fine extensions and dynamic nature enable microglia to survey the entire brain parenchyma for potential infection or cell damage and, upon activation, they release cytokines and proceed to the phagocytosis of cell debris or infectious micro-organisms. The experimental activation of microglia by the administration of the bacterial endotoxin lipopolysaccharide (LPS) has been shown to decrease adult neurogenesis, by specifically inhibiting the proliferation or the survival of the new cells (Ekdahl et al., 2003; Monje et al., 2003; Fujioka and Akema, 2010). These effects may be mediated by the release of cytokines such as IL-6, TNFα, IL-1β, since these molecules show an inhibitory effect on adult neurogenesis *in vitro* or *in vivo* (Vallieres et al., 2002; Monje et al., 2003; Keohane et al., 2010; Kohman and Rhodes, 2013).

Recently, the role of microglia in absence of lesion or inflammation has started to raise interest. Indeed, recent studies suggest that resting microglia may be involved in the regulation of physiological mechanisms such as dendritic spine maintenance (Paolicelli et al., 2011) or the homeostasis of the neurogenic niche by the removal of apoptotic newborn cells (Sierra et al., 2010). Furthermore, neural progenitor cells (NPCs) regulate microglia function *in vitro*, by the secretion of growth factors (Mosher et al., 2012). However, the role of microglia on adult neurogenesis in physiological conditions remains unclear.

Here, we examined the correlation between the amount of microglia in the hippocampus and the proliferation of adult neural stem cells, in physiological conditions. To increase the variability in progenitor proliferation, we used aging and voluntary exercise, as these parameters are known to decrease and increase neurogenesis respectively (Kuhn et al., 1996; van Praag et al., 1999, 2005; Encinas et al., 2011; Kempermann, 2011).

## Materials and methods

### Ethics statement

This study was carried out in strict accordance with the recommendations in the Guidance for the Care and Use of Laboratory Animals of the National Institutes of Health. All experimental protocols were approved by the Swiss animal experimentation authorities (Service de la consommation et des affaires vétérinaires, Chemin des Boveresses 155, 1066 Epalinges, Switzerland, permit number: 2301). Every effort was made to minimize the number of animals used and their suffering.

### Experimental animals

All animals used for this study were adult male mice. GFAP-GFP mice were a kind gift from the laboratory of Helmut Kettenmann (Max-Delbruck center, Berlin, Germany) (Nolte et al., 2001). They express the green fluorescent protein (GFP) under the control of the human glial fibrillary acidic protein promoter (GFAP). At the beginning of the experiment, the first group of mice was 6-week-old and the second group 7.5-month-old. Runner mice were housed for 2 weeks in standard cages equipped with a running wheel (Fast-Trac; Bio-Serv, USA) and were allowed free access to the running wheel. Non-runner mice were housed in similar, adjacent cages without running wheel. All mice were housed in a 12-h light/dark cycle and controlled temperature of 22°C. Food and water were available *ad-libitum*.

### BrdU administration

Immediately at the end of the running period, all mice were injected intraperitoneally with the DNA replication marker, Bromodeoxyuridine (BrdU, Sigma-Aldrich, Buchs, Switzerland), at doses of 100 mg/kg in saline, 3 times at 2-h intervals. Two hours after the last injection, mice were sacrificed and the number of BrdU cell was counted to assess cell proliferation (Mandyam et al., 2007; Taupin, 2007; Yang et al., 2011; Gao and Chen, 2013).

### Tissue collection and preparation

At the end of the experiment, mice received a lethal dose of pentobarbital (10 mL/kg, Sigma-Aldrich, Buchs, Switzerland) and were perfusion-fixed with 50 ml of 0.9% saline followed by 100 mL of 4% paraformaldehyde (Sigma-Aldrich, Switzerland) dissolved in phosphate buffer saline (PBS 0.1M, pH 7.4). Brains were then collected, postfixed overnight at 4°C, cryoprotected 24 h in 30% sucrose and rapidly frozen. Coronal frozen sections of a thickness of 40 μm were cut with a microtome-cryostat (Leica MC 3050S) and slices were kept in cryoprotectant (30% ethylene glycol and 25% glycerin in 1X PBS) at –20°C until processed for immunostaining.

### Immunohistochemistry

Immunochemistry was performed on 1-in-6 series of section. Sections were washed 3 times in PBS 0.1 M. BrdU detection required formic acid pretreatment (formamide 50% in 2X SSC buffer; 2X SSC is 0.3 M NaCl and 0.03 M sodium citrate, pH 7.0) at 65°C for 2 h followed by DNA denaturation for 30 min in 2 M HCl for 30 min at 37°C and rinsed in 0.1 M borate buffer pH 8.5 for 10 min. Then, slices were incubated in blocking solution containing 0.3% Triton-X100 and 15% normal serum normal goat serum (Gibco, 16210-064) or normal donkey serum (Sigma Aldrich, D-9663), depending on the secondary antibody in PBS 0.1 M. Slices were then incubated 40 h at 4°C with the following primary antibodies: mouse monoclonal anti-BrdU (1:250, Chemicon International, Dietikon, Switzerland), rabbit anti-Ki-67 (1:200, Abcam, ab15580), goat anti-DCX (1:500, Santa Cruz biotechnology, sc-8066), rabbit anti-GFAP (1:500, Invitrogen, 180063), rabbit anti-Tbr2 (1:200, Abcam, ab23345), goat anti-Iba1 (1:200, Abcam, ab5076), mouse anti-MHC-II (1:200 Abcam, ab23990). The sections were then incubated for 2 h in either of the following secondary antibodies: goat anti-mouse Alexa-594 (1:250, Invitrogen), goat anti-rabbit Alexa-594 (1:250, Invitrogen), donkey anti-goat Alexa-555 (1:250, Invitrogen). 4,6 diamidino-2-phenylindole (Dapi) was used to reveal nuclei.

### Cell culture

Adult NPCs expressing the red fluorescent protein (RFP) are a kind gift from the laboratory of Fred Gage (Salk Institute, San Diego, USA). They were isolated from the DG of adult Fisher 344 rats and cultured as previously described (Palmer et al., 1997). Microglia and astrocyte primary culture were purified from postnatal day 3 rats. Cerebral cortices, including the hippocampus, were mechanically triturated for homogenization and seeded onto poly-D-lysine coated 75 cm^2^ flasks in Dulbecco's Modified Eagle Medium (DMEM) glutamax (Invitrogen, USA), 10% normal calf serum with penicillin/streptomycin (Invitrogen, USA). Cells were grown for 5–7 days in a humidified 5% CO2 incubator at 37°C. At confluence, flasks were shaken at 250 rpm on an orbital shaker for 2 h to separate microglia from astrocytes. Detached microglia were seeded in poly-D-lysine coated 6-well microplates in culture medium supplemented with 30% astrocyte conditioned medium, to enhance the survival of microglia (personal communication from Dr. Romain Gosselin, University of Lausanne). Different numbers of microglia or astrocytes were plated on coated 12 mm coverslips in a 24-well culture plate (20,000, 40,000, 60,000 cells per well). Three hours later, a fixed number of RFP-expressing NPCs (20,000 cells per well) were plated on the same culture wells, at a (NPCs/Glia) ratio of 1/0, 1/1, 1/2, 1/3. Three wells per condition were used. Ninety-six hours after plating, cells were fixed with 4% paraformaldehyde for 20 min, briefly washed and immunostained for Iba1 or GFAP and mounted.

### Cell number quantification

All images were acquired using a confocal microscope (Zeiss LSM 710 Quasar Carl Zeiss, Oberkochen, Germany). The total number of immunoreactive cells was estimated throughout the entire granule cell layer using stereological sampling, as previously described (Thuret et al., 2009), between –1.3 to –2.9 mm from the Bregma. For each animal, a 1-in-6 series of sections was stained with the nucleus marker DAPI and used to measure the volume of the granule cell layer. The granule cell area was traced using Axiovision (Zeiss, Germany) software and the granule cell volume was determined by multiplying the traced granule cell layer area by the thickness of the corresponding section and the distance between the sections sampled (240 μm). For all mice analyzed in this study, no difference of volume was found between groups [One-Way ANOVA, *F*_(3, 15)_ = 0.29, *p* = 0.82], (data not shown). All cells were counted blind with regard to the mouse status. The number of labeled cells was counted for each section, in the entire thickness of the granular cell layer of the DG with a 40x objective. RGL cells and cells expressing BrdU, Ki-67, DCX or Tbr2 were counted in an area containing the granule cell layer and the subgranular zone, whereas cells expressing Iba1, MHC-II and GFAP (Figures 2A,D,F) were counted in an area including the sub-granular cell layer, the granule cell layer, the molecular layer and the hilus. Cells expressing Iba1 (Figure 2B) were also counted in the primary somatosensory cortex (S1).

For *in vitro* cell quantification, images were acquired using confocal microscopy. The numbers of RFP-expressing NPCs, Iba1-expressing microglia and GFAP-expressing astrocytes were counted in 4 selected fields, systematically placed in the same positions relative to the coverslips' edges. The total number of cells per selected field was divided by the total area of the selected fields to obtain an average cell density per well that was then multiplied by the total surface area of the coverslip to obtain an estimate of the total number of cells per coverslip. This number of cells was then compared to the number of cells that were plated in the wells to obtain a percentage of increase in NPCs number.

### Statistical analysis

Hypothesis testing was two-tailed. All analyses were performed using JMP10 software. First, Shapiro-Wilk tests were performed on each dataset to test for distribution normality. The distribution was normal for all data. To test for possible interaction between the 2 groups (Aging and Running) a Two-Way ANOVA was performed. When an interaction was found, a One-Way ANOVA test was performed, if no interaction was found, we analyzed each variable independently using a Two-Way ANOVA. All analyses were followed by a *post-hoc* Tukey HSD test. In order to test linear correlation, Spearman correlation test was performed. Results are expressed by the Spearman correlation coefficient (ρ) and the *p*-value. Data is presented as mean ± standard error of the mean (SEM).

## Results

### Effect of aging and running on cell proliferation and immature neurons

**Figure 1:**
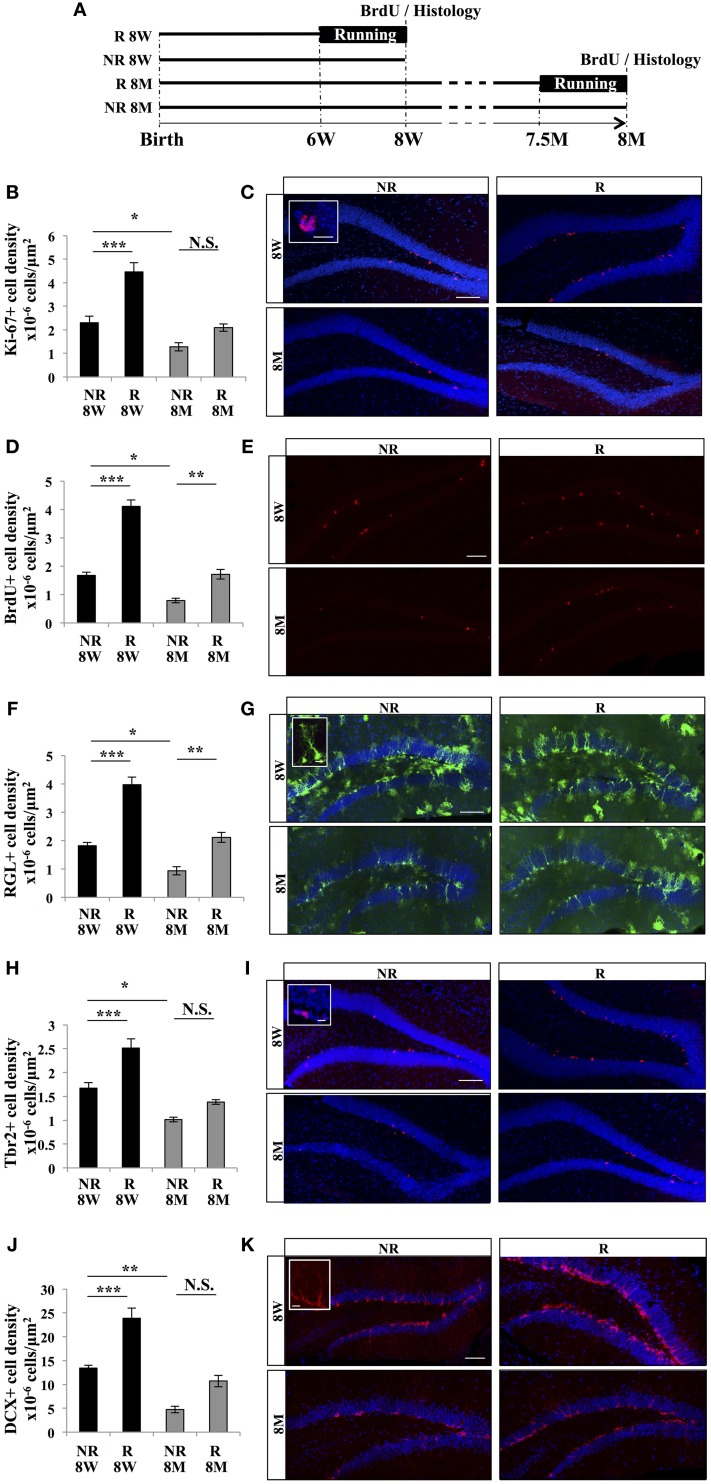
**Effect of running and aging on cell proliferation in the dentate gyrus. (A)** Experimental timeline. **(B)** Histogram of the density (cells/μ m^2^) of Ki-67–expressing cells in the granule cell layer of the dentate gyrus of 8-week-old animals (8 W) and 8-month-old animals (8 M) housed with (R), or without (NR) a running wheel. **(C)** Confocal maximal projection micrographs of hippocampal sections immunostained for Ki-67. Inset: Higher magnification confocal micrograph of a Ki-67-expressing cell. **(D)** Histogram of the density of BrdU-positive cells in the granule cell layer of the dentate gyrus. **(E)** Confocal maximal projection micrographs of hippocampal sections immunostained for BrdU. **(F)** Quantification of the density of RGL cells in the granule cell layer of the dentate gyrus. **(G)** Confocal maximal projection micrographs of hippocampal sections. Inset: Higher magnification confocal micrograph of a RGL cell. **(H)** Histogram showing the density of Tbr2-expressing cells in the granule cell layer of the dentate gyrus. **(I)** Confocal maximal projection micrographs of hippocampal sections immunostained for Tbr2. Inset: Higher magnification confocal micrograph of a Tbr2-expressing cell. **(J)** Histogram of the density of DCX-immunolabeled cells in the granule cell layer of the dentate gyrus. **(K)** Confocal micrographs of hippocampal sections immunostained for DCX. Inset: Higher magnification confocal micrograph of a DCX-immunolabeled group of cells. Blue: Dapi staining. Animals, *n* = 4 per group. Scale bars: 100 μm, insets 10 μm, *post-hoc* Tukey HSD test ^*^p < 0.05; ^**^p < 0.01; ^***^p < 0.001; NS: *p* > 0.05. Each value represents the mean ± SEM.

We tested the effect of running and aging on GFAP-GFP mice (Nolte et al., 2001), which are commonly-used mice models for the examination of adult neurogenesis and enable the identification of stem cells (Huttmann et al., 2003; Ehninger and Kempermann, 2008). Sixteen GFAP-GFP mice were divided in 4 experimental groups: young adult mice (8 weeks of age; 8 W) and older mice (8 months of age; 8 M), that were previously housed individually in standard cages in presence (R) or absence (NR) of a running wheel for 2 weeks (4 mice per group). At the end of the 2 weeks period, all mice received 3 intraperitoneal injections of BrdU (100 mg/kg) at 2 h intervals and were sacrificed 2 h after the last BrdU injection (Figure 1A). Brains were sectioned and immunostained for BrdU and markers for adult neurogenesis and microglia. We first assessed cell proliferation in the granule cell layer of the DG. A Two-Way ANOVA test revealed that there is interaction between aging and running for Ki-67 marker [*F*_(1, 12)_ = 5.74; *p* < 0.05]. The density of Ki-67-expressing cells was significantly decreased by aging [Figures 1B,C, One-Way ANOVA *F*_(3, 15)_ = 22.65; *p* < 0.001. *Post-hoc* Tukey HSD test: NR 8 W vs. NR 8$\dot{\text{M}}$ *p* < 0.05] and increased by running [One-Way ANOVA *F*_(3, 15)_ = 22.65; *p* <0.001. *Post-hoc* Tukey HSD test NR 8 W vs. R 8 W *p* < 0.001, NR 8 M vs. R 8 M *p* = 0.24].

Similarly, there is interaction between aging and running for BrdU marker [Two-Way ANOVA *F*_(1, 12)_ = 17.6; *p* < 0.01]. The density of BrdU-expressing cells was significantly decreased with aging [Figures 1D,E, One-Way ANOVA *F*_(3, 15)_ = 62.19; *p* < 0.001. *Post-hoc* Tukey HSD test: NR 8 W vs. NR 8 M, *p* < 0.05] and increased by running [One-Way ANOVA *F*_(3, 15)_ = 62.19; *p* < 0.001. *Post-hoc* Tukey HSD test: NR 8 W vs. R 8 W *p* < 0.001, NR 8 M vs. R 8 M *p* < 0.01]. Thus, cell proliferation was increased by voluntary running and reduced by aging.

In the DG, proliferative cells are divided in 2 main cell populations: the type 1, radial glia-like (RGL) stem cells and the type 2, TAPs. RGL cells were identified by the expression of GFP, their nucleus located in the subgranular zone and a radial process extending through the granule cell layer and branching into the molecular layer (Huttmann et al., 2003; Mignone et al., 2004; Kriegstein and Alvarez-Buylla, 2009; Beckervordersandforth et al., 2010). They expressed the self-renewal factor Sox2 (data not shown). For RGL, Two-Way ANOVA test showed that there is interaction between aging and running [*F*_(1, 12)_ = 5.3; *p* < 0.05]. The density of RGL cells was significantly decreased by aging (Figures 1F,G, One-Way ANOVA *F*_(3, 15)_ = 35.81; *p* < 0.001. *Post-hoc* Tukey HSD test: NR 8 W vs. NR 8 M *p* < 0.05] and increased by running [One-Way ANOVA *F*_(3, 15)_ = 35.81; *p* < 0.001. *Post-hoc* Tukey HSD test: NR 8 W vs. R 8 W *p* < 0.001, NR 8 M vs. R 8 M *p* < 0.01]. TAPs were identified by immunostaining against Tbr2 (Hodge et al., 2008). Similarly, there is interaction between aging and running for Tbr2 marker [Two-Way ANOVA *F*_(1, 12)_ = 5.2 *p <* 0.05]. The density of Tbr2-expressing cells was decreased by aging [Figures 1H,I, One-Way ANOVA *F*_(3, 15)_ = 27.32; *p* < 0.001. *Post-hoc* Tukey HSD test: NR 8 W vs. NR 8 M *p* < 0.05] and increased by running [One-Way ANOVA *F*_(3, 15)_ = 27.32; *p* < 0.001. *Post-hoc* Tukey HSD test NR 8 W vs. R 8 W *p* < 0.001, NR 8 M vs. R 8 M *p* = 0.2]. Thus, running increased and aging decreased the density of stem and progenitor cells.

Finally, we examined the effect of aging and running on immature neurons identified by immunohistochemistry against doublecortin (DCX). A Two-Way ANOVA test revealed that there is no interaction between aging and running for DCX marker [*F*_(1, 12)_= 2.21; *p* = 0.16]. Although, The density of DCX-expressing cells decreased with aging [Figures 1J,K, Anova *F*_(1, 12)_ = 52.90; *p* < 0.001. *Post-hoc* Tukey HSD test: NR 8 W vs. NR 8 M *p* < 0.01] and increased with running [*F*_(1, 12)_ = 29.97; *p* < 0.001. *Post-hoc* Tukey HSD test NR 8 W vs. R 8 W *p* < 0.001, NR 8 M vs. R 8 M *p* = 0.06]. Together, these results indicate that the density of proliferative cells, cell proliferation and the density of immature neurons decreased with aging and increased with voluntary running.

### Effect of aging and running on microglia

**Figure 2:**
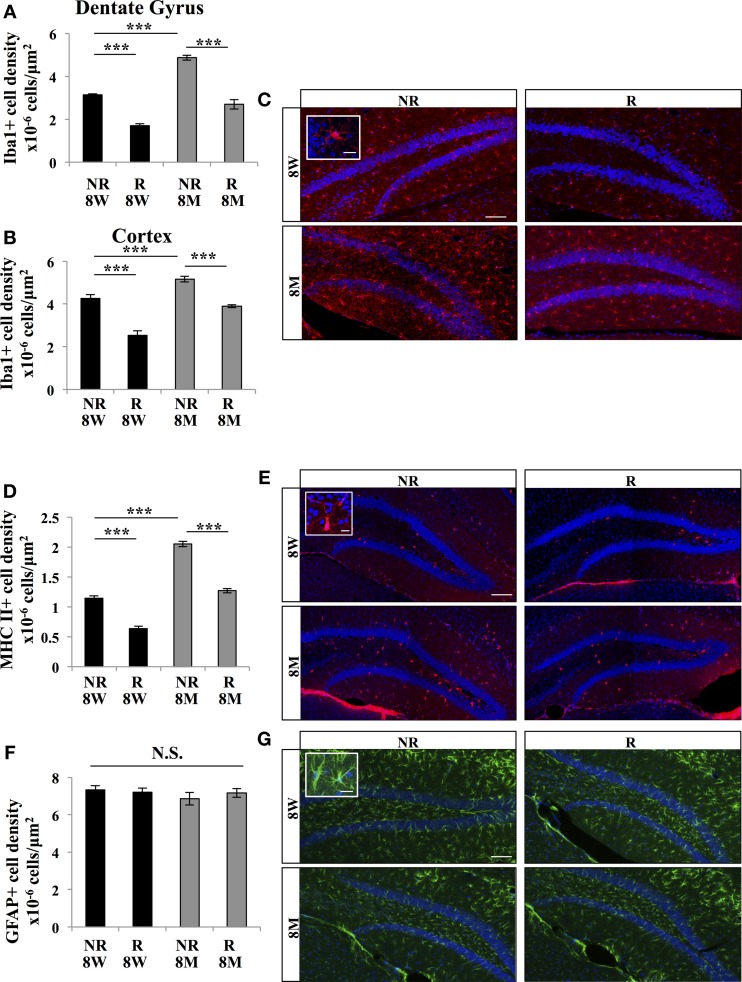
**Effect of running and aging on the density of microglia. (A)** Histogram showing the density (cells/μ m^2^) of Iba1-expressing cells in the dentate gyrus (including hilus, granule cell layer and molecular layer). **(B)** Histogram of the density of Iba1-expressing cells in the cortex. **(C)** Confocal maximal projection micrographs of hippocampal sections immunostained for Iba1. Inset: Higher magnification confocal micrograph of an Iba1-immunolabeled cell. **(D)** Histogram of the density of MHC II-expressing cells in the dentate gyrus. **(E)** Confocal maximal projection micrographs of hippocampal sections immunostained for MHC II. Inset: Higher magnification confocal micrograph of a MHC II-immunolabeled cell. **(F)** Histogram of the density of GFAP-expressing astrocytes in the dentate gyrus of mice for each experimental condition. **(G)** Confocal maximal projection micrographs of hippocampal sections immunostained for GFAP. Inset: Higher magnification confocal micrograph of a GFAP-immunolabeled cell. Blue: Dapi staining. Animals: *n* = 4 per group. Scale bars: 100 μm, insets 10 μm. *post-hoc* Tukey HSD test: N.S.: *p* > 0.05; ^***^p < 0.001. Each value represents the mean ± SEM.

Next we examined the effect of aging and running on microglia, using the immunomarker Iba1. Iba1-expressing cells displayed an oval cell body and numerous ramified processes (Figures 2A–C). A Two-Way ANOVA revealed that there is interaction between aging and running for Iba1 marker [*F*_(1, 12)_ = 5.65; *p* < 0.05]. The density of Iba 1-expressing cells in the DG was decreased by running [Figures 2A–C, One-Way ANOVA *F*_(3, 15)_ = 75.94; *p* < 0.001. *Post-hoc* Tukey HSD test: NR 8 W vs. R 8 W *p* < 0.001, NR 8 M vs. R 8 M *p* < 0.001] and increased by aging [One-Way ANOVA *F*_(3, 15)_ = 75.94; *p* < 0.001. *Post-hoc* Tukey HSD test: NR 8 W vs. NR 8 M *p* < 0.001]. To examine whether the effect of running and aging on microglia was restricted to the hippocampus, we measured microglia in the primary somato-sensory cortex of all mice. Contrary to what we observed in the DG, in the primary somato-sensory cortex, there was no interaction between aging and running in the level of Iba1 marker [Two-Way ANOVA *F*_(1, 12)_ = 2.1; *p* = 0.17]. Anova showed that running increased [Figure 2B, *F*_(1, 12)_ = 92.47; *p* < 0.001. *Post-hoc* Tukey HSD test: NR 8 W vs. R 8 W *p* < 0.001, NR 8 M vs. R *p* < 0.001] whereas aging decreased the density of microglia in the cortex [*F*_(1, 12)_ = 52.49; *p* < 0.001. *Post-hoc* Tukey HSD test: NR 8 W vs. NR 8 M *p* < 0.001].

To test whether the expression of the antigen presentation protein MHC II was also regulated by aging and running, we immunostained brain slices for MHC II. A Two-Way ANOVA revealed that there is interaction between aging and running for MHC II marker [*F*_(1, 12)_ = 8.08 *p* < 0.01]. The density of MHC II-expressing cells was decreased by running [Figures 2D,E, One-Way ANOVA *F*_(3, 15)_ = 145.19; *p* < 0.001. *Post-hoc* Tukey HSD test: NR 8 W vs. R 8 W *p* < 0.001, NR 8 M vs. R 8 M *p* < 0.001] and increased by aging [One-Way ANOVA *F*_(3, 15)_ = 145.19, *p* < 0.001. *Post-hoc* Tukey HSD test: NR 8 W vs. NR 8 M *p* < 0.001]. Finally, we examined whether astroglia was similarly affected by running or aging by immunostaining against GFAP. The density of GFAP-expressing astrocytes was constant throughout all conditions [Figures 2F,G; Two-Way ANOVA *F*_(3, 12)_ = 0.49, *p* = 0.69], indicating that the effect of aging and running was specific to microglia.

### Correlation between microglia and adult neurogenesis

**Figure 3:**
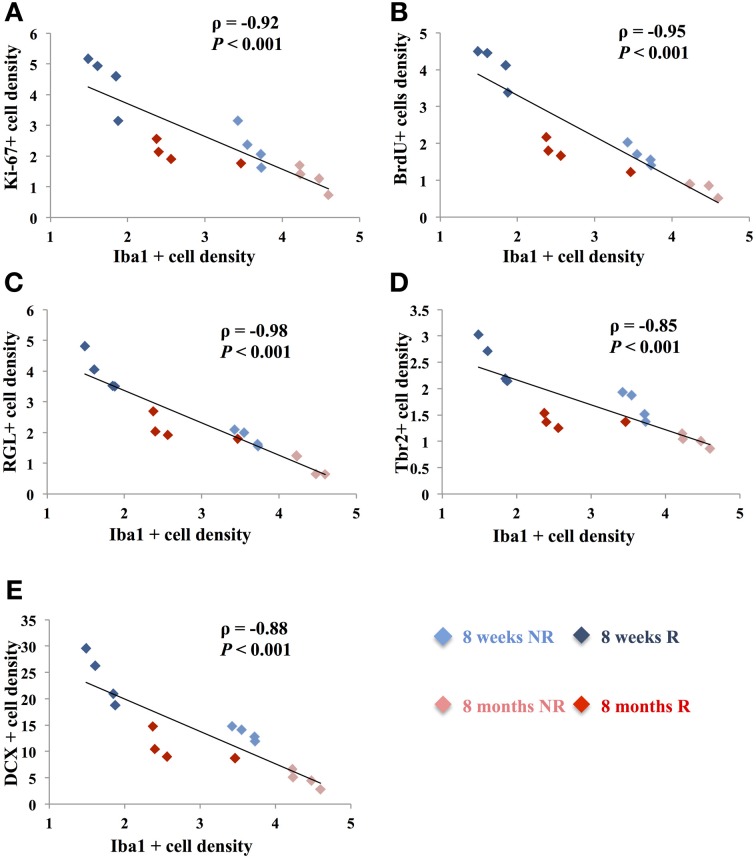
**Correlation between cell proliferation and Iba1-expressing microglia. (A–E)** Plots showing the density of Ki-67-expressing cells **(A)**, BrdU-labeled cells **(B)**, RGL cells **(C)**, Tbr2-expressing cells **(D)** and DCX-expressing cells **(E)**, as a function of the density of Iba1-expressing cells, in the dentate gyrus. Each point represents the density of cells for one animal. Spearman's correlation test was used to study the correlation, ρ: coefficient of Spearman; *p*: value of significance.

The opposite effects of running and aging on microglia and adult neurogenesis suggest that microglia and adult neurogenesis may be inversely correlated. We therefore plotted, for each mouse, the number of Iba-expressing microglia and the number of cells expressing markers for neurogenesis. The number of Iba1-expressing microglia was inversely correlated with the number of Ki-67-expressing cells (Figure 3; ρ = −0.92, *p* < 0.001), RGL cells (ρ = −0.98, *p* < 0.001), Tbr2-expressing cells (ρ = −0.85, *p* < 0.001), BrdU-immunolabeled cells (ρ = −0.95, *p* < 0.001), and DCX-expressing cells (ρ = −0.88, *p* < 0.001). Similarly, the number of MCH II-expressing microglia inversely correlated with the number of Ki-67-expressing cells (Data not shown; ρ = −0.95, *p* < 0.001), RGL cells (ρ = −0.92, *p* < 0.001), Tbr2-expressing cells (ρ = −0.97, *p* < 0.001), BrdU-immunolabeled cells (ρ = −0.94, *p* < 0.001), and DCX-expressing cells (ρ = −0.97, *p* < 0.001). These results indicate that microglia density was inversely correlated with stem/progenitor cell proliferation.

**Figure 4:**
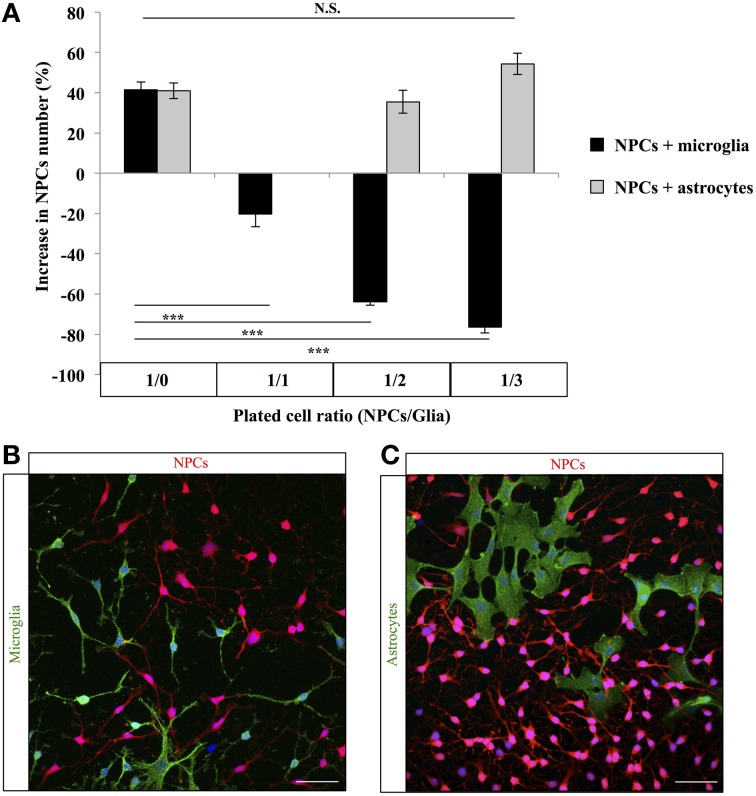
**Effect of microglia on progenitor cells *in vitro*. (A)** Histogram of the change in NPCs number when cultured with microglia or astrocytes (% from the number of plated cells). *N* = 3 culture wells per group. **(B)** Confocal maximal projection micrographs of NPCs-microglia co-cultures. **(C)** Confocal maximal projection micrographs of NPCs-astrocytes co-cultures. N.S.: *p* > 0.05, ^***^p < 0.001. Each value represents the mean ± SEM.

To test the possibility that microglia number could directly affect stem/progenitor cell proliferation, we co-cultured microglia and NPCs. We plated a constant number of NPCs with an increasing proportion of microglia and 4 days later, we counted the number of remaining NPCs. The number of NPCs significantly decreased with the increasing proportion of co-cultured microglia [Figure 4; One-Way ANOVA *F*_(6, 20)_ = 82.58, *p* < 0.001; 1/0 vs. 1/1 *post-hoc* Student's *t*-test *p* < 0.001, 1/0 vs. 1/2 *post-hoc* Student's *t*-test *p* < 0.001, 1/0 vs. 1/3 *post-hoc* Student's *t*-test *p* < 0.001]. To test whether this effect was specific to microglia, we repeated this experiment with increasing proportion of astrocytes instead of microglia. In contrast to microglia, astrocytes did not decrease the number of NPCs in co-cultures [One-Way ANOVA *F*_(6, 20)_ = 82.58, *p* < 0.001; 1/0 vs. 1/2 *post-hoc* Student's *t*-test *p* = 0.53, 1/0 vs. 1/3 *post-hoc* Student's *t*-test *p* = 0.13]. Thus, increasing microglia density inhibited NPCs growth and/or survival *in vitro* and resulted in a corresponding decrease in NPCs number.

### Discussion

In this study, we exploited the physiological variations of adult neurogenesis induced by voluntary running and aging to examine the correlation between microglia and adult hippocampal neurogenesis in absence of inflammatory stimulus. In line with previous studies, we found that aging decreased cell proliferation and the number of immature neurons whereas voluntary running had inverse effects (Kuhn et al., 1996; van Praag et al., 1999, 2005; Encinas et al., 2011; Kempermann, 2011). In addition, we found that the decrease in RGL cells observed with aging (Encinas et al., 2011), was reversed by voluntary running, suggesting that running may induce the symmetrical division of RGL cells and restore their population. Strikingly, in both conditions, the amount of microglia but not of astroglia was strongly inversely correlated with all measured parameters of neurogenesis. Similarly, *in vitro*, co-cultures of NPCs with an increasing proportion of microglia but not of astroglia reduced the number of NPCs after 4 days. Together, these results indicate that, in physiological conditions, microglia and neurogenesis are inversely correlated and suggest that microglia may inhibit adult neurogenesis by directly acting on stem/progenitor cells.

These observations are in line with previous experiments showing that, in inflammatory conditions induced by epilepsy, ischemia or LPS injection, microglia undergo dramatic changes in their morphological and cytokine expression pattern and inhibit adult neurogenesis (Monje et al., 2002; Ekdahl et al., 2003; Liu et al., 2007; Kohman et al., 2012). Similarly, aging is associated with a reduced neurogenesis, mild, chronic inflammation and increased microglia proliferation that can be attenuated by voluntary running (Kohman et al., 2012). In these experimental paradigms, inflammatory microglia is believed to directly inhibit neurogenesis, since anti-inflammatory treatments restore neurogenesis (Ekdahl et al., 2003; Monje et al., 2003; Liu et al., 2007; Kohman and Rhodes, 2013) and the exercise-induced increase and the age-dependent decline in neurogenesis are mediated by microglia *in vitro* (Vukovic et al., 2012).

However, in our study, we did not induce inflammation and in absence of activation, microglia has been reported to promote neurogenesis: In adult rats, the increased neurogenesis induced by environmental enrichment was accompanied by an increase in microglia number, whereas immunodeficient mice had impaired neurogenesis (Ziv et al., 2006). Similarly, voluntary running was shown to increase neurogenesis (van Praag et al., 1999) and microglia proliferation (Ehninger and Kempermann, 2008; Encinas et al., 2011), without inducing changes in the total number of microglia cells or in their activation state (Encinas et al., 2011). *In vitro* too, microglia was shown to promote the proliferation of co-cultured NPCs, an effect believed to be mediated by released factors, since the pro-neurogenic effect was mimicked by microglia-conditioned medium (Morgan et al., 2004; Aarum et al., 2003; Walton et al., 2006; Nakanishi et al., 2007). Thus, it is surprising that in our study, we observed an inverse correlation between microglia number and neurogenesis, in absence of inflammatory stimulus, in particular in running and non-running young mice. This discrepancy may partly result from differences in housing conditions, diet or animal strain. Indeed, while our study examined transgenic mice in the FVB/N background, the Ziv study used adult male rats (Ziv et al., 2006) and the Olah study used adult male C57Bl/6 mice (Olah et al., 2009). FVB/N mice are known to have reduced cell proliferation in the adult DG, as compared to C57Bl/6 mice (Schauwecker, 2006) and may therefore respond differently to microglia regulation, or have increased basal inflammatory state. However, the absence of inflammatory stimulus in our *in vivo* experiments as well as the morphological aspect of microglia suggestive of a resting state, indicate no overt inflammation in GFAP-GFP mice. Furthermore, the observation that microglia reduced the number of NPCs *in vitro* supports the idea of a direct inhibition of progenitor cell proliferation by microglia. Finally, in a recent study, we found that doxycycline treatment decreased microglia cell number and increased neurogenesis and cell proliferation in C57Bl/6 mice (Sultan et al., 2013), suggesting that environmental factors and housing conditions may interfere with the effect of microglia on adult neurogenesis, rather than mouse strain.

However, correlation does not imply causality and the signaling between microglia and neurogenesis in the adult healthy brain remains unclear. Although activated microglia can directly inhibit neurogenesis by releasing a number of pro-inflammatory cytokines (Monje et al., 2003; Nakanishi et al., 2007), external factors such as voluntary running may act on neurogenesis independently from microglia. For example, the exercise-induced increase in neurogenesis depends on peripheral VEGF (Fabel et al., 2003) that acts directly on NPCs of the hippocampus (Fournier et al., 2012). Running can also affect microglia by reducing the expression of pro-inflammatory cytokines such as TNF-α, and increasing the expression of the anti-inflammatory cytokines such as IL-1ra (Pervaiz and Hoffman-Goetz, 2011) or the chemokine CX_3_CL1, that induces a neuroprotective microglia phenotype and promotes neurogenesis (Vukovic et al., 2012). Inversely, gene expression analysis in the hippocampus showed that running induced the transcription of genes involved in inflammation, including genes related to MHC I (β 2-microglobulin, H2-D1) and elements of the complement system (C4A, C3, C1q) or in the inflammatory response (COX-2, CX3C), suggesting that running may increase inflammation (Tong et al., 2001; Kohman et al., 2011).

Depending on its activation state, microglia may have opposite effects on adult neurogenesis and it is likely that in the same brain, pro-neurogenic and anti-neurogenic microglia co-exist, with a different response to external stimulus, such as voluntary running and housing conditions. The combined action of external factors and microglia state may then result in unexpected effects on adult neurogenesis. Clearly, further experiments will be necessary to elucidate the regulation of neurogenesis by microglia and the role played by environmental factors such as stress and activity on both microglia and neurogenesis. This knowledge is crucial for the understanding of the regulation of adult neurogenesis by the neurogenic niche as well as for the therapeutic use of neural stem cells in inflammatory context such as lesions or neurodegenerative disorders.

### Author contributions

Conceived and designed the experiments: Elias Gebara, Sebastien Sultan and Nicolas Toni. Performed the experiments: Elias Gebara, Sebastien Sultan and Jacqueline Kocher-Braissant Analyzed the data: Elias Gebara and Sebastien Sultan Wrote the paper: Elias Gebara, Sebastien Sultan and Nicolas Toni.

### Conflict of interest statement

The authors declare that the research was conducted in the absence of any commercial or financial relationships that could be construed as a potential conflict of interest.
